# Microfluidics: Rapid Diagnosis for Breast Cancer

**DOI:** 10.1007/s40820-015-0079-8

**Published:** 2016-01-08

**Authors:** Satvinder Panesar, Suresh Neethirajan

**Affiliations:** BioNano Laboratory, School of Engineering, University of Guelph, Guelph, ON N1G 2W1 Canada

**Keywords:** Mircofluidics, MiRNA, Biomarkers, Diagnostic technology, Breast cancer

## Abstract

Breast cancer affected 1.7 million people worldwide in 2012 and accounts for approximately 23.3 % of all cancers diagnosed in women. The disease is characterized by a genetic mutation, either inherited or resulting from environmental factors, that causes uncontrollable cellular growth of breast tissue or adjacent tissues. Current means of diagnosing this disease depend on the individual analyzing the results from bulky, highly technical, and expensive equipment that is not globally accessible. As a result, patients can go undiagnosed due to a lack of available equipment or be over-diagnosed due to human error. This review attempts to highlight current means of diagnosing breast cancer and critically analyze their effectiveness and usefulness in terms of patient survival. An alternative means based on microfluidics biomarker detection is then presented. This method can be considered as a primary screening tool for diagnosing breast cancer based on its robustness, high throughput, low energy requirements, and accessibility to the general public.

## Introduction

Consider how illnesses are presently diagnosed. A patient develops some symptoms, they visit their doctor, and their doctor runs tests to identify the ailment. Next, either the doctor figures out the problem and prescribes some treatments, or the patient is sent to a specialist for further tests. At the visit to the specialist, more tests are conducted until a diagnosis is reached; then, the patient is treated based on this diagnosis. For a majority of illnesses, this method of diagnosing a patient is generally successful; however, in the case of cancer, once symptoms begin to manifest, it may already be too late.

**Fig. 1:**
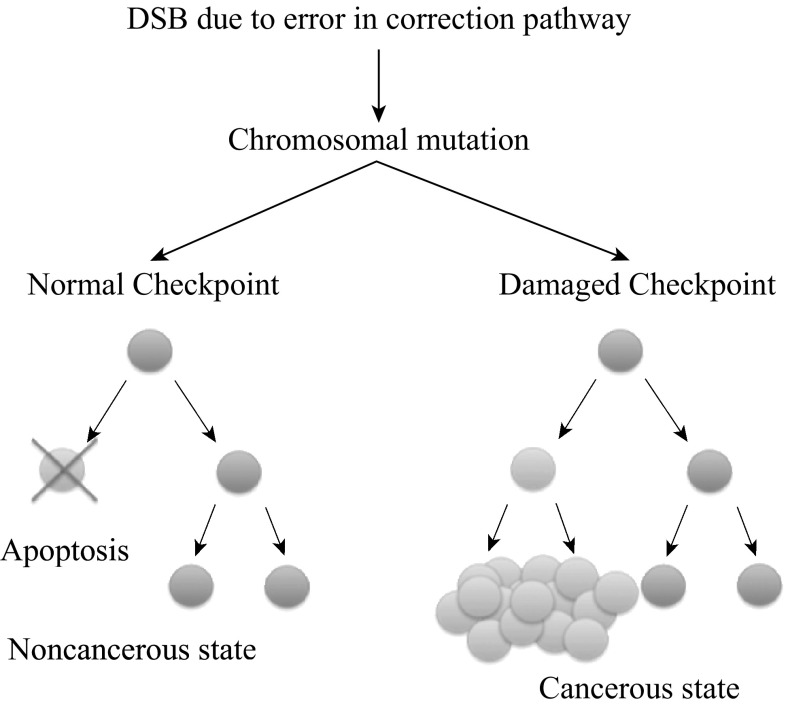
Schematic depicts a DSB leading to a genetic mutation. The DSB causes damage to the DNA strand and leads to a cancerous state (*right*). However, during correction, cellular death occurred, and a cancerous state was avoided (*left*)

Age, lifestyle, genetics, nutrition, and stress all play their respective roles in the development of cancers [1, 2]. As a person ages, becomes more sedentary [3–6] and gains excess weight [7–9], the rate of diagnosis of serious life-threatening diseases such as cancer is amplified [10–13]. The Centers for Disease Control and Prevention (CDC) report that malignant neoplasms are second only to heart diseases as a worldwide cause of deaths [14]. Globally, the proportion of deaths attributed to malignant neoplasms has risen from 12.49 % of all deaths in 2002, when they ranked third in worldwide causes of death, to 23.3 % today [14, 15]. The development of a malignant neoplasm is a complex process and depends on many factors, such as genetic susceptibility, damage to the DNA, and even the location in the body where the neoplasm starts. As the malignant neoplasm advances into its later stages, survival rates drop exponentially [16–19]. Preventative measures such as exercising, eating a healthy diet, avoiding stress, and managing weight can be taken to reduce the risk of developing malignant neoplasms; however, these measures do not eliminate the effect of genetic predispositions or mutations that may occur during an individual’s life. Figure 1 shows how an error can cause cancer due to a double-strand break (DSB) in the DNA.

In many cancer cases, an error such as the example in Fig. 1 can lead to an extremely detrimental state for a patient. However, early detection can play a major role in patient survival, especially in cases involving breast cancer. The patient survival rate drops exponentially as the stages of cancer progress. Patients’ survival rates go down from a 100 % 5-year survival after first diagnosis in stage one to 93 % in stage two, 72 % in stage three, and 22 % in stage four [20]. Therefore, the key to surviving malignant neoplasms is the early detection when preventative measures fail.

If we can reevaluate how we diagnose breast cancer and develop a more proactive means of diagnosing the disease before it develops into a serious condition rather than after symptoms present themselves, we can take steps toward making diagnostic medicine a much more effective tool for doctors to use.

This review examines breast cancer, specifically the biomarkers that aid in diagnosing the disease, the methods currently associated with breast cancer detection, and the reasons why microfluidics should be the first choice in breast cancer detection to increase survival rates through early detection.

## Breast Cancer

**Fig. 2:**
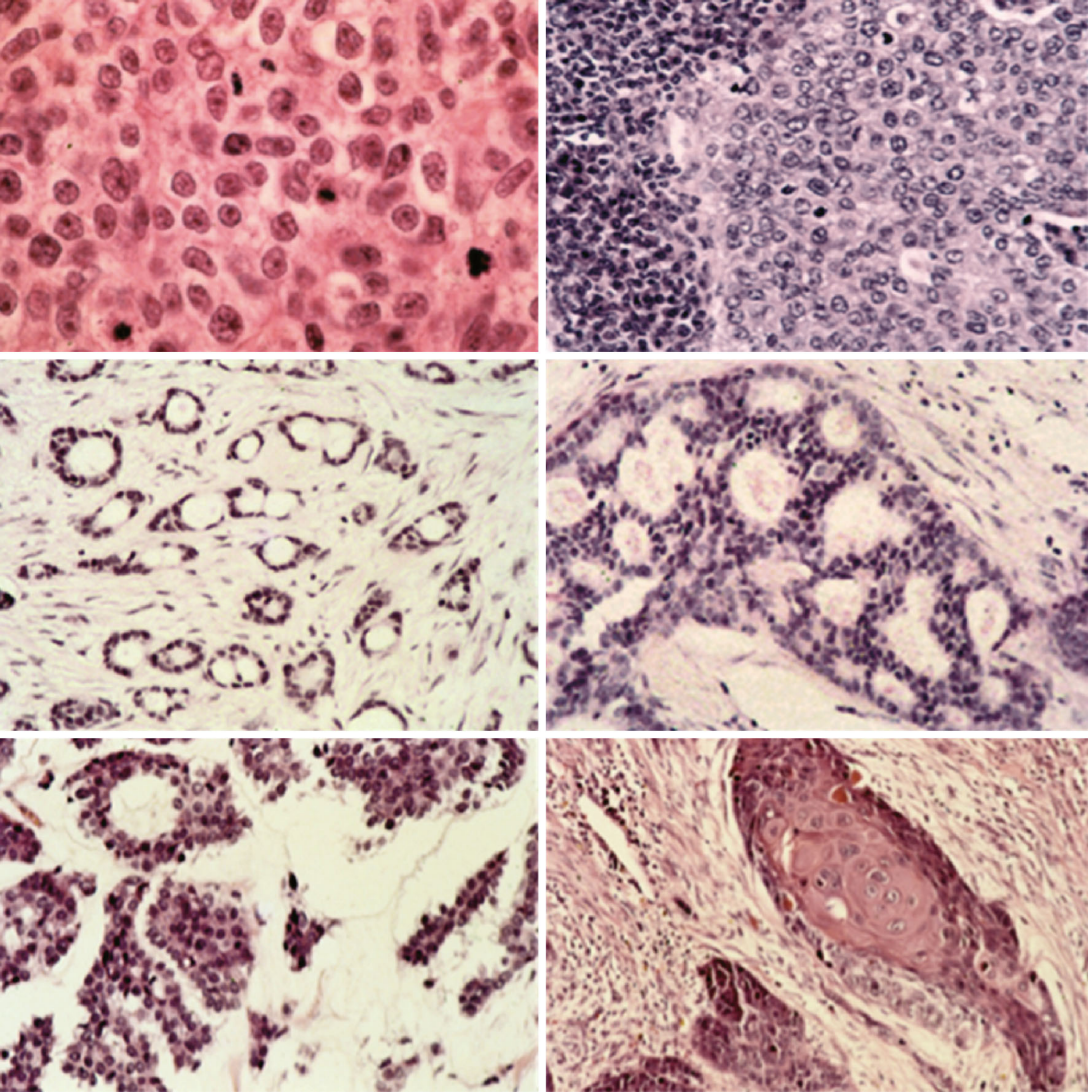
Histological variance in breast cancer subtypes. Images from top *left* to *bottom right*: Invasive carcinoma of no special type, medullary, tubular, cribriform, mucinous, and squamous metastatic breast cancer. Image taken from Breast: Ductal Carcinoma [117]

Breast cancer is a category of malignant neoplasms that occur in or around the breast tissue, and it is a type of adenocarcinoma. This broad-scale classification encompasses many types of malignant neoplasms that can occur in or around the breast tissue. Each type of neoplasm owns specific type of disease with morphological, molecular, and clinical variations [21]. Figure 2 shows how different subtypes of breast cancers demonstrate different histological characteristics.

The type of breast cancer that a person may develop is dependent on the site of the neoplasm. The two most common histological types of breast cancer are lobular and ductal carcinomas (LCIS and DCIS, respectively) [21–23]. These two types account for 80–95 % of breast cancer cases, while the remaining cases are rarer forms of breast cancer such as acinic-cell carcinoma, adenoid cystic carcinoma, comedo carcinoma, glycogen-rich clear cell carcinoma, invasive apocrine carcinoma, invasive papillary and micropapillary carcinomas, lipid-rich carcinoma, medullary carcinoma, metaplastic carcinoma, mucinous carcinoma, neuroendocrine carcinoma, sebaceous carcinoma, and secretory carcinoma [22, 24]. In addition to these variations of histological types of breast cancer, there are hormonal variations in breast cancer such as estrogen receptor-positive (ER^+^) or -negative (ER^−^), progesterone receptor-positive (PR^+^) or -negative (PR^−^), and human epidermal growth factor receptor 2-positive (HER2^+^) or -negative (HER2^−^) cancers. These variations correspond to different types of treatment that can be offered for breast cancer.

### Stages of Breast Cancer

Prior to treatment, an individual’s breast cancer is placed within a “stage” ranging from 0 to IV. Staging breast cancer helps to document the severity of the disease and whether the malignant neoplasms have metastasized to other parts of the body or remained in their local tissue. To determine where the neoplasms are, a physician may conduct a lymph node biopsy using a sentinel lymph node biopsy (SLNB) or other tests such as a bone scan, computed tomography (CT) scan, magnetic resonance imaging (MRI), or positron emission tomography (PET) scan. A SLNB involves the identification and removal of sentinel lymph nodes as well as examination to determine whether neoplastic cells are present. A negative biopsy means that neoplastic cells have not yet made their way to the sentinel lymph nodes, whereas a positive biopsy indicates that neoplastic cells are present in sentinel lymph nodes and may potentially be present in regional lymph nodes and other organs. Once the neoplastic cells are located in the body, and it is determined whether they have metastasized, the cancer is assigned a stage (Table 1). The staging of breast cancer is based on the TNM classification of tumors, as set by the American Joint Committee on Cancer (AJCC), and is shown in Table 2 and explained in Table 1. The TNM scale is used to differentiate the tumor size (T), the lymph-node involvement (N), and whether any metastasization has occurred (M).

**Table 1:** Breast cancer staging and explanations for each stage given [26]

Stage	Explanation
0	Benign neoplasm, typically referred to as ‘carcinoma in situ’ (in original place). Usually found in ductal carcinoma in situ (DCIS), lobular carcinoma in situ (LCIS) and pagets disease of the nipple Non-invasive neoplasm, typically harmless and surgically removable
IA	Malignant neoplasms that have not yet spread to other parts of the body or the lymph nodes. Diameter of 20 mm or less. Localized to breast tissue
IB	A malignant neoplasm that has begun to spread to lymph nodes in the breast tissue. The size of this neoplasm in lymph nodes is around 0.1 to 2.0 mm. Breasts may or may not show signs of neoplasms. If neoplasms do exist, size does not exceed 20 mm
IIA	Malignant, invasive neoplasm that is either: (1) Present in fewer than four axillary lymph nodes and has not been found in the breast (2) Present in breast tissue, is less than 20 mm in size and has spread to less than four axillary lymph nodes, or (3) Present in the breast tissue, has a diameter between 20 and 50 mm and has not yet spread to any axillary lymph nodes
IIB	Malignant, invasive neoplasm that is either (1) Present in the breast tissue, has a diameter between 20 and 50 mm and has spread to less than four axillary lymph nodes, or (2) Present in the breast tissue, has a diameter greater than 50 mm, and has not yet spread to any axillary lymph nodes
IIIA	Malignant, invasive neoplasm that is either (1) Present in the breast tissue, has a diameter less than 20 mm and has spread to more than four lymph nodes, but is less than nine lymph nodes, or (2) Present in the breast tissue, has a diameter greater than 50 mm and neoplastic breast tissue is found in the lymph nodes, or (3) Present in the breast tissue, has a diameter greater than 50 mm and neoplastic breast tissue is found in the lymph nodes under the arm or at/around the breastbone
IIIB	A malignant, invasive neoplasm of any size that has metastasized to the chest wall or breast skin, showing signs of swelling, ulcers, inflammation, and has spread to less than nine regional lymph nodes
IIIC	Malignant, invasive neoplasm that is either 1) Not present in the breast tissue or it is a neoplasm of any size in the breast tissue, has metastasized to the chest wall or breast skin, shows signs of swelling, ulcers, and inflammation, and has spread to ten or more lymph nodes under the arm, or 2) Not present in the breast tissue or it is a neoplasm of any size in the breast tissue and has metastasized to the lymph nodes in the collarbone region, or 3) Not present in the breast tissue or it is a neoplasm of any size in the breast tissue and has metastasized to lymph nodes under the arm or in the breastbone region
IV	Malignant invasive neoplasm that has metastasized to other parts of the body

**Table 2:** TNM Chart classification for breast cancer based on the AJCC [27]

T	N		M
T0	NX		MX
Tis	N0	N0 (i +)	M0
		N0 (mol +)	
T1	N1	N1mi	M1
		N1a	
		N1b	
		N1c	
T2	N2	N2a	
		N2b	
T3	N3	N3a	
		N3b	
		N3c	
T4			

Although the two most common breast cancers, DCIS and LCIS, own different names, they originate from the same place. Both begin in the terminal duct-lobular unit (TDLU), which connects the lobes to the ducts in the breast. To differentiate DCIS from LCIS, the growth patterns of the neoplastic tissue as well as the cytological phenotypes of the tissue are examined. During examination, DCIS typically presents with calcification on a mammogram, is typically unilateral (occurring in or on only a single breast), and can form a myriad of different structures under microscopic observation [21]. LCIS is bilateral, dyscohesive under a microscope and is typically not found on a mammogram [21] (Table 3).

**Table 3:** Explanation table for TNM rating for breast cancer based on the AJCC [27]

Rating	Explanation
TX	Tumor cannot be assessed
T0	No evidence of a tumor
Tis	Carcinoma In Situ, typically associated with DCIS, LCIS or Pagets disease of the nipple
T1	Tumor diameter of 2 cm or less
T2	Tumor diameter greater than 2 cm but less than 5 cm
T3	Tumor diameter greater than 5 cm
T4	Tumor of any diameter invading the chest wall or skin
NX	Unable to assess lymph nodes
N0(i +)	Small levels of cancer cells found in the underarm lymph nodes. Cancer cells less than 200 cells and smaller than 0.2 mm
N0(mol +)	Cancer cells not visible in the underarm lymph nodes, but detected via RT-PCR
N1mi	Micrometastasis in 1 to 3 lymph nodes under the arm. Cancer cells have a diameter of 2 mm or less and at least 200 cancer cells
N1a	Cancer spread to 1 to 3 lymph nodes under the arm; one area has cancer cells with a diameter of 2 mm or greater
N1b	Spread to internal mammary lymph nodes, lymph nodes not enlarged. Detected via sentinel lymph node biopsy
N1c	Both N1a and N1b
N2a	Cancer found in 4 to 9 lymph nodes and one location has cancer cells larger than 2 mm
N2b	Cancer spread to one or more internal mammary lymph nodes with enlargement in the nodes
N3a	Either Cancer in 10 or more axillary lymph nodes; one area has cancer cells with a diameter greater than 2 mm OR Cancer spread to lymph nodes under clavicle; one area has cancer cells with a diameter greater than 2 mm
N3b	Cancer found in axillary lymph nodes with a diameter greater than 2 mm and the internal mammary lymph nodes have enlarged OR Cancer spread to 4 or more axillary lymph nodes; cancer cells have a diameter of 2 mm or greater and are found on internal mammary lymph nodes on a sentinel lymph node biopsy
MX	Metastasis cannot be determined
M0	No distant cancer cells found on imaging equipment
M1	Cancer spread to distant organs

## Biomarkers

When a patient develops breast cancer, many physiological changes occur within the body. Protein expressions and levels are altered; genes become mutated, causing their expression levels to change; microRNA (miRNA) expression levels change; and physical changes, such as lumps, inflammation, and changes in skin, may also manifest themselves [25–29]. Due to the complexity of breast cancer and the way the human body operates, no single gene, protein, or miRNA test has yet been developed that definitively proves whether a patient has breast cancer. Multiple tests are often required, and many biomarkers are examined before any conclusions can be drawn about the patient’s conditions and prognosis. Here, we will look at different biomarkers involved with breast cancer, starting with genetic markers.

### Genetic Markers

**Fig. 3:**
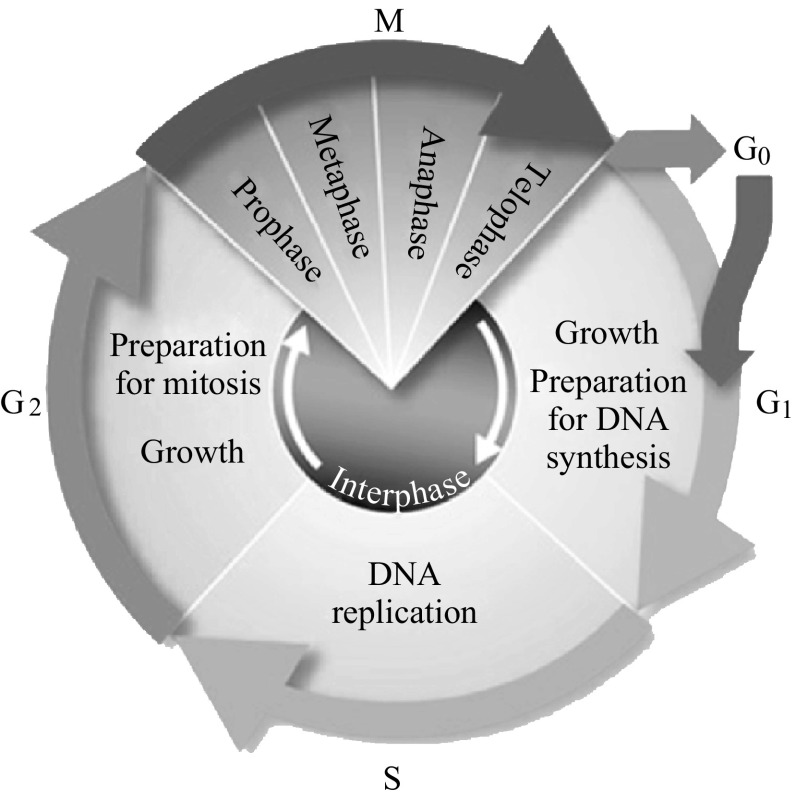
Cell cycle diagram [118]. Beginning at the G1 phase, cells observe environmental conditions and await stimuli to begin the replication process. During the G1 phase, when the conditions are correct, the cells begin the replication process by synthesizing the required RNAs and proteins. In the S phase, the chromosomal DNA is replicated, and in the G2 phase, the cells prepare for mitosis. Mitosis occurs in the M phase

Genetic markers are DNA mutations that can serve as biomarkers to indicate the presence of a disease such as breast cancer. Changes in regulation of protein expression, loss of function of a cell, or even uncontrollable division can occur if mutations in cellular DNA go uncorrected. Here, some common genetic markers associated with breast cancer are examined. Most markers addressed in this section play their roles in the cell cycle; an illustration of the cell cycle process is shown in Fig. 3.

#### *BRCA1/2*

The most common genetic marker examined for potential breast cancer cases is the breast cancer susceptibility 1 (BRCA1) and 2 (BRCA2) genes. The BRCA1/2 genes are responsible for creating the BRCA1 and BRCA2 proteins, respectively. They are located on the long arm of chromosome 17 at position 21 and the long arm of chromosome 13 at position 12.3, respectively. The BRCA1 protein is responsible for DNA repair, signal transduction, and tumor suppression [30–34]. BRCA1/2 proteins are also responsible for repairing DSBs in the DNA sequence [30]. BRCA1/2 utilize two methods for DSB repairs: homologous recombination (HR) repair and non-homologous end-joint repair (NHEJ) [30, 35, 36]. Currently, researchers believe that tumorigenesis occurs when both the BRCA1 and BRCA2 genes are damaged or lost, leading to a lack of proteins available to repair damaged DNA [37].

**Fig. 4:**
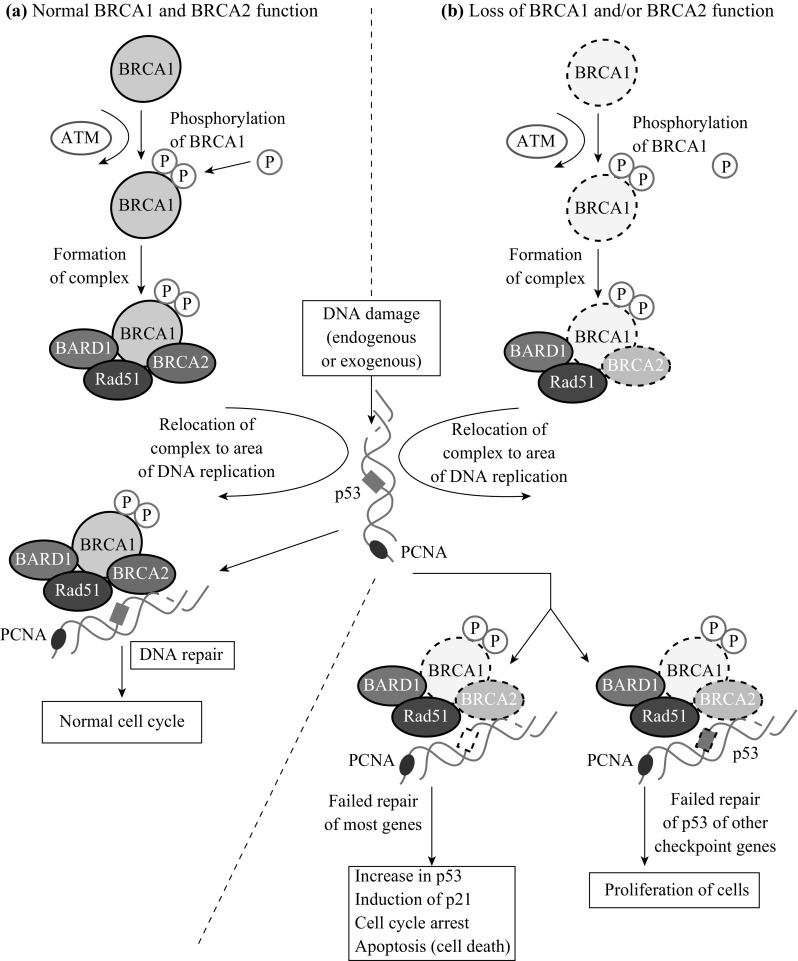
HR repair of DSB by BRCA1 and BRCA2. Image taken from Kiyotsugu et al. [119]

The BRCA1 gene belongs to a different family than the BRCA2 gene. The BRCA1 gene belongs to the RING-type zinc finger family (RNF), whereas the BRCA2 gene belongs to the Fanconi anemia complementation group (FANC). The BRCA1 protein is also reported to have the ability to crosslink repair-damaged DNA strands [38]. Long and Walter suggest that BRCA1 modifies halted replication at the DNA fork terminal in order to antagonize a protein known as Ku70/Ku80 heterodimer (a protein responsible for NHEJ repair of DNA strands) to prepare the strands for binding with ubiquitylated FANCD2 (a group of proteins in the FANC group, similar to BRCA2) [38]. This suggests that BRCA1 attempts to call upon BRCA2 proteins to help with DNA repair to suppress tumors. BRCA1 is able to repair DSB with HR repair via its C-terminal, as depicted in Fig. 4. This motif of the protein is responsible for interacting with RNA polymerase and CtIP to maintain proper DNA structure [39]. Mutations in this region affect BRCA1′s ability to repair DNA and also hinder its ability as a tumor suppressor.

#### PALB2

PALB2, a partner and localizer of BRCA2, is another gene commonly associated with breast cancer. It codes for the PALB2 proteins, whose function is tumor suppression. This gene enlists the help of BRCA2 and RAD51 (discussed below) in DNA breaks via HR repair. PALB2 localizes and accumulates BRCA2 for DSB via HR repair and is also responsible for localizing the BRCA2-RAD51 complex for DNA repair [40]. As the name suggests, PALB2 enlists the support of BRCA2 (and BRCA1) to create a BRCA1-PALB2-BRCA2 (BPB) complex that provides HR repair [40]. PALB2 creates the BPB complex by interacting with BRCA1 via its own N-terminal coiled-coil domain and with BRCA2 via its own C-terminal WD40 domain [41]. The PALB2 gene is located on the short arm (*p*) of chromosome 16 at position 12.2. It is suggested by Rahman et al. that PALB2 mutations may be associated more with male breast cancer cases than with female cases because of the involvement of BRCA2 [42]; however, this needs to be investigated further. Buisson et al. found that, while traditionally it was thought that BRCA2 and PALB2 regulated HR repair through regulation of the RAD51 protein, PALB2 is also important for Pol η (polymerase η) localization as well as DNA polymerization activity [41]. Mutations of PALB2 hinder its ability to properly synthesize DNA, leading to breast cancer due to mutations in the DNA. DSBs resulting from a PALB2 mutation are not properly repaired, and the cells can potentially enter a cancerous state [41].

#### BRIP1

BRCA1 interacting protein C-terminal helicase 1 (BRIP1) is a gene that encodes for the BRIP1 protein. BRIP1, located on the long arm of chromosome 17 at position 22.2, interacts with BRCA1 to form a bound complex that repairs DSBs in DNA to prevent damage and a potentially cancerous state. This is similar to how PALB2 operates on BRCA2. Mutations in this gene are responsible for germline mutations that can induce cancer. The BRIP1 gene is also implicated in the Fanconi anemia (FA) DNA repair pathway, a pathway responsible for repairing DSBs in DNA to prevent other genetic conditions [40]. BRIP1 functions by maintaining chromosomal stability via its interaction with the C-terminal in BRCA1 [39, 43]. DNA damage is evaluated at the G_2_ cell-cycle checkpoint, and, if needed, any corrections can be made [40]. Mutations in this gene hinder its ability to properly check for DNA mutations through messenger RNA (mRNA) involvement [40].

#### RAD51 recombinase (RAD51)

**Fig. 5:**
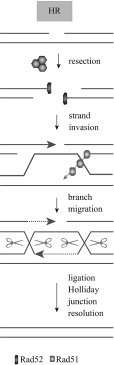
HR Repair of DNA with RAD51. RAD51 binds to the 3′ end of DNA, initiating DNA polymerase and repairing the damaged sites. Image taken from Khanna and Jackson [120]

RAD51 is a gene that codes for the RAD51 protein, which is responsible for the repair of DSBs in DNA via the HR repair mechanism. Figure 5 explains how RAD51 is involved with DNA repair. It is located on the long arm of chromosome 17 at position 22, which is very close to BRCA1, BRIP1 and RAD51C. RAD51 is known to interact with BRCA2 to generate a complex that is responsible for DNA repair [40]. RAD51 regulates BRCA1 and BRCA2 in HR and localizes BRCA1 in the nuclear foci during the S and G_2_ phases of the cell cycle [40]. RAD51 simultaneously interacts with BRCA2’s BRC repeats to repair damaged DNA [44, 45]. The BRC repeat is a set of 35 amino acids (aa) repeated eight times, creating a distinct motif. This is where RAD51 joins with BRCA2 to repair DSBs in the DNA into single strand breaks (SSBs) [45]. Mutations in the RAD51 gene interfere with its ability to localize BRCA2 to repair sites where DSBs exist. This allows DNA damage to get coded and could potentially lead to a cancerous state. Mutations that can arise in this way are called ‘missense mutations’. They affect RAD51’s ability to bind BRCA2. It was also reported by Woditschka et al. that overexpression of the RAD51 protein leads to brain metastases from breast cancer [46]. Another paper highlights the point that regulation of RAD51 needs to be exact, as both too much and too little can cause serious consequences for the body, such as cancer metastasization and increased resistance to DNA repair [47].

#### RAD51 Homolog C (RAD51C)

RAD51C belongs to the RAD51 class of genes involved in the recombinant repair of DNA. It is one of five paralogs of RAD51 and is responsible for DNA repair via HR [48]. RAD51C is involved with two separate protein complexes, the RAD51B-RAD51C–RAD51D–XRCC2 complex and the RAD51C–XRCC2 complex [48]. RAD51C creates a complex for HR repair with both PALB2 and BRCA2. RAD51C is located on the long arm of chromosome 17 at position 22.

#### BRCA1-Associated RING Domain 1 (BARD1)

BARD1 is a gene that encodes for the human protein BRCA1-associated RING domain protein 1. BARD1 is located on the long arm of chromosome 2 at position 35. It contains a RING finger, as does BRCA1, and when working together they exhibit ubiquitin ligase activity [49]. The function of BARD1 in the body’s response to breast cancer remains relatively unknown other than that it is present in the nucleus with BRCA1 during DNA repair. However, research by Dr. Kaufmann suggests that it is used to stabilize the BRCA1 C-terminal complex in order to repair DSBs [50]. The utilization of BARD1 by BRCA1 in DNA repair is shown in Fig. 4. BARD1 mutations are considered a high cancer risk and need further examination, as their exact function after mutation is relatively unknown.

#### Phosphate and Tensin Homolog (PTEN)

PTEN is a gene that encodes for the PTEN protein, a protein which is found in several different cancers [51–54]. It is found in nearly all tissues in the body and is responsible for tumor suppression [55, 56], apoptosis [57, 58], angiogenesis [59] and many other functions [60–62]. PTEN is located on the long arm of chromosome 10 at position 23.3. PTEN is involved in the 1-phosphatidylinositol 3-kinase pathway (PI3K), which is responsible for cell proliferation and survival. In breast cancer, this results in PTEN down-regulating PI3K, causing cellular arrest during the G1 phase of the cell cycle [54]. In general, loss of PTEN in breast cancer results in a poor prognosis for the patient [54].

### Receptor Markers

Receptor markers are markers that interact with tumor cells. If a tumor cell binds a corresponding receptor, it is considered receptor positive, and if it does not, it is considered negative. When determining whether the cancer is receptor positive or negative, an oncologist will run a test where the cells are stained with a dye. Out of the sample of cells, the dye will stain some cells but not others. The stained cells carry the receptor being measured. From this, the oncologist determines what percentage of cells is stained to assess whether receptor-based treatment will be successful. The ratio of stained to unstained cells indicates how many of the cancer cells express the corresponding receptors. In addition to the proportion of cells stained, the saturation of the stain is also measured, where a deeper stain indicates a greater intensity of the receptor. From this test, the oncologist determines whether the cancer is receptor positive or negative. This, however, is not an exact science and will vary based on the oncologist conducting the test. For example, recovering 15 out of 100 cells with estrogen receptors may be considered a receptor-negative result for one lab but a positive result for another. A score of zero, however, always corresponds to a hormone-receptor negative result, and in such a case, hormonal therapy would be ineffective. Below, there is a discussion of the three receptors for which testing is currently available.

#### HER2

HER2 is a gene that codes for the HER2 protein, which manifests as a receptor on breast cells. Over-expression of this gene results in an increase of HER2 receptors on breast cells, which in turn causes the breast cells to grow and divide in a cancerous manner. This HER2-positive form of breast cancer is very aggressive.

#### Estrogen Receptor (ER) and Progesterone Receptor (PR)

ER- and PR-based breast cancers occur when breast cancer tumor cells contain ERs or PRs. These receptors stimulate tumor growth in size and number when there is an increase in the concentration of these hormones in the blood. If a tumor does not contain estrogen or progesterone receptors, it is considered to be ER−/PR−. These two hormones are produced by the ovary and fluctuate in concentration based on the menstrual cycle.

### MicroRNA

MicroRNAs (miRNAs) are a class of non-coding RNAs that are 22 nucleotides long whose function is to silence RNA and affect post-transcriptional regulation of genes [63–65]. There are over 200 different known miRNAs, and their expression changes in response to cancer, making them an excellent candidate for detection and diagnosis of the disease. It has also been reported that miRNAs exhibit involvement with tumorigenesis [66–68]. Because of their relationship with many diseases, including breast cancer, miRNAs are being studied more and more. New information is being revealed on protein expression profiles, new pathways in disease progression, cellular growth, differentiation, and cellular processes, as well as protein and RNA silencing as further research on this class of RNAs is conducted. MiRNAs are formed in the body with the help of different proteins. DNA is transcribed by messenger RNA (mRNA), which is then transcribed by RNA polymerase II into pri-miRNA [69]. MiRNAs are cleaved from pre-miRNA precursors by cytoplasmic RNase III Dicer [64]. Then, two strands are made, and one strand degrades while the other becomes the mature miRNA [69]. The effects of miRNA on breast cancer are still not fully understood, but their expression profiles help with the diagnosis of breast cancer. MiRNAs can also be easily accessed from whole blood, not just serum or plasma like other biomarkers, making them a very powerful diagnostic tool that requires minimal invasiveness. It has also been reported that miRNA levels are not biased (varying) based on whether the condition is in situ, is invasive (IDC, ILC), has a particular intrinsic subtype (Luminal A, Luminal B, HER2-enriched and claudin-low) or its HER2 status [70]. Because miRNA levels will be elevated for breast cancer types, miRNAs are a potentially sensitive and robust marker for the diagnosis of breast cancer.

#### Let-7a

The let-7a miRNA belongs to the let-7 family of miRNAs. This family was one of the first families of miRNAs discovered and is typically poorly expressed or completely deleted in breast cancer cases [71]. Let-7a belongs to a class of cells called tumor-initiating cells (TICs), which can divide asymmetrically as well as undergo self-renewal [71]. Researchers are currently exploring the mechanisms involved in up- and down-regulation of let-7a cells in breast cancer.

#### MiR-10b

MiR-10b is overexpressed in many metastatic cancers, including breast cancer, and is known to initiate tumor invasion in vivo, and cell migration and invasion in vitro [63, 71]. Ma et al. reported that miR-10b levels were 50-fold higher in metastatic breast cancer cells compared to non-metastatic breast cancer cells [63]. Interestingly, it was reported by Sethi et al. that elevated miR-10b levels caused breast cancer to metastasize to the brain, but after metastasizing to the brain, miR-10b levels would decrease [72]. Another study showed that levels of miR-10b were found to be higher in patients with ER^−^ breast cancer compared to ER^+^ [70]. Additional research on mi-R10b’s function is required to determine the mechanisms and pathways that cause it to operate. While it is a key miRNA for breast cancer, a more concrete understanding of its role in metastatic cancer and different breast cancer subtypes is needed before it can be utilized in clinical applications.

#### MiR-21

MiR-21 is over-expressed in breast cancer cells and is up-regulated in breast cancer tumor cells compared to normal, healthy breast cells [71]. Frankel et al. reported that miR-21 regulates the human protein programmed cell death 4 (PDCD4) and has links to the protein p53. Inhibiting levels of miR-21 increase the levels of PDCD4 3.5-fold [73]. MiR-21 expression also correlates with breast cancer prognosis. Patients with higher levels of miR-21 have a significantly worse prognosis compared to patients with lower levels of miR-21 (85.54 % survival for low expression versus 45.90 % survival for high expression after a five year period) [65]. It was also observed that patients with ER^−^ breast cancer expressed higher levels of miR-21 compared to patients with ER^+^ breast cancer, similar to miR-10b [70].

#### MiR-155

MiR-155 is found to be up-regulated in breast cancer cells that have not metastasized [71]. Gasparini et al. show in their findings that the over-expression of miR-155 in breast cancer cells inhibits the effects of RAD51 in triple negative breast cancer. MiR-155 targets the untranslated region of the 3′ in RAD51, resulting in a decreased efficiency of RAD51 at repairing DSB through HR [74]. This process, however, is beneficial to ionizing radiation therapy and the treatment of breast cancer. MiR-155 can be considered a breast cancer metastasis promoting gene because of its effect on breast cancer.

#### MiR-195

MiR-195 is a key miRNA in breast cancer detection. It was found to be elevated in breast cancer cells compared to healthy cells and was reported by Heneghan et al. to exhibit a 19.25-fold change between the two groups [70]. The same study reported a detection of 85.5 % sensitivity and 100 % specificity in the miRNA as well as an increase in tumor miRNA expression with later stages of breast cancer [70].

## Diagnostic Tests for Breast Cancer Detection

When diagnosing breast cancer, physicians have a large repertoire of tests at their disposal. Some tests are simple and require no advanced training or equipment, while other tests require highly sensitive equipment, large amounts of energy, and a great deal of time to run. Physicians can draw samples from a patient for analysis, including tissue biopsies, blood work or urine work, or they can conduct radiography tests to image what is happening under the skin. Currently, however, the use of microfluidics as a diagnostic tool is under-valued. Microfluidics offer many versatile means of detection, low energy requirements, and high throughput, and accuracy, as well as minimal time requirements.

### Mammogram

Mammograms are the most commonly used imaging technique when looking for breast cancer. They utilize ionizing radiation at very low energy levels (30 kVp) to generate images. The purpose of the use of mammograms is to find tumors before physical lumps can be felt. This is done in an attempt to diagnose cancer at its early stages so that preventative measures can be taken, and the chance of mortality from the disease is lowered. However, mammography has led to over-diagnosis and treatment of patients who in actuality never had any breast tumors [75]. One limiting factor in the use of mammography is the resolution at which they can generate images. Because they generate images from X-rays, they have a finite maximum resolution, with the result that tumors under the size of 0.01 nm are not visible on a mammogram. This, however, does not affect their use because most tumors in early cases of breast cancer are in the mm range. Mammograms need to be able to differentiate the tumor tissue in the breasts from the soft tissue, which is very similar [76]. In a mammogram, there are five distinct characteristics that make up the images: the artifacts, the blur, the contrast sensitivity, the geometry, and the noise of the image. Each of these five components is adjusted to generate images that provide the user with the best visibility of the inner breast tissue. The mechanism behind mammograms involves an X-ray tube made from either molybdenum or molybdenum and rhodium to produce x-ray radiation. The beam is emitted from a focal spot between 0.1 and 0.3 mm and through a filter. The filter decreases any unnecessary exposure of the patient to X-ray radiation. The X-rays pass through the breast tissue and onto a grid, which is used to absorb scattered radiation and improve the image of the scan. Below the grid is a digital and film receptor. This receptor takes X-rays and generates an image of the mammogram scan [76]. The material for the anode as well as the kV value are selected based on the size and density of the breast tissue; typical kV values range between 24 and 32 kV [76]. The higher the kV value, the more the x-rays will penetrate the tissue and create a higher-contrast image. The major limitations of mammograms include the size of the equipment, the energy required to run the equipment, and the need for a trained individual to run and interpret the results. While they are a powerful tool, mammograms require many resources to operate and, as stated above, can result in over-diagnosis and false positives as well as false negatives.

### CT Scan

CT scan is another x-ray-based technique to diagnose breast cancer. Like mammography, it provides no insight into the genetics involved with the cancer or the hormone receptor status. It is merely used to detect signs of breast cancer. CT scan has five specific image characteristics: blurring and detail, visual noise, artifacts, tomographic slices, and contrast sensitivity [77]. CT scan differs from mammograms in requiring less involvement of a trained technician and providing much faster results [77]. However, one drawback of CT scan is the need for both ionizing radiation and intravenous contrast material for image generation. The contrast material can have adverse effects on the patient and, in rare circumstances, can result in death. A CT scan works by rotating the unit around a patient while moving up or down over the region to be scanned. The scanning methods for a CT scan include step and scan, and helical or spiral scanning. Step and scan works by rotating around one slice of the body, then moving to another slice, and rotating again. Helical or spiral scanning works by rotating around the body while the body is moved up or down. In the scan, three values are of interest: the pitch, the width, and the distance per revolution. The width corresponds to the width of the beam, which determines how wide the scanning area will be. The distance per revolution is how far the body (or machine) is moved per revolution, and the pitch is the distance moved per revolution per width. A faster pitch results in a quicker scan, but the resolution might not be as high [77]. In addition to single beam scans, a multiple beam scan can also be performed. This allows the scan to run faster, and data are overlapped to generate complete images of the scan area. Like mammography equipment, CT scanners are very bulky and require a great deal of resources to operate. While powerful, CT scanning cannot be used in the field to diagnose breast cancer.

### MRI

MRI is a powerful imaging technique that utilizes magnetic fields and radio frequencies to generate images of breast tissue. The MR image is dependent on a few factors, including the proton density of the tissue, longitudinal and transverse relaxation times of the tissue, vascular flow of the fluid, perfusion of the fluid, diffusion of the fluid, and chemical spectroscopy [77]. The proton density of the tissue relates to image generation, so tissue with higher proton densities will show up brighter and with more intensity than a tissue with lower density. The longitudinal and transverse relaxation times pertain to how long it takes the tissue to go from a magnetized state to a normal state and vice versa [77]. These two times are denoted T1 and T2. This produces contrast between images because healthy and tumorous tissues differ in their relaxation times. Vascular flow, perfusion, and diffusion of the blood create contrast within the image to help distinguish flowing regions from tissue regions as well as sediments or artifacts within the flow of fluids. Lastly, the spectroscopic chemical fluid adds additional contrast to help distinguish different features within the image from one another. These factors combine to emit a radio frequency that generates the image. The images are generated when a patient is placed inside the magnetic field of the machine and the magnetic nuclei within their tissues emit a radio frequency [77]. Image generation and variation is dependent on the tissue and fluid characteristics. Therefore, in a breast MRI, a tumor would show up differently from normal healthy tissue because of its different characteristics. When the image is being generated, the tissue goes through a series of magnetizations. Similar to a pulse occurring over a period of time, the tissue is subjected to a series of magnetizations over a period of time, resulting in different intensities of radio frequency emission [77]. While this is a powerful tool, MRI is very costly to operate, requires a great deal of energy, is not mobile in anyway, and is subject to the interpretation of oncologists. While they are highly trained individuals, there is always the chance for human error, and false negatives and positives in the images.

### Ultrasound

Ultrasound imaging is a much more basic tool compared to the other tools discussed. It employs the use of a transducer to emit ultrasonic sound waves into the tissue. This method has no adverse effects on the patient and can provide images immediately. It can be portable, is easy to operate and can create detailed images; however, it does not provide definitive proof of a tumor, as the images produced could represent calcified stones.

### PCR

**Fig. 6:**
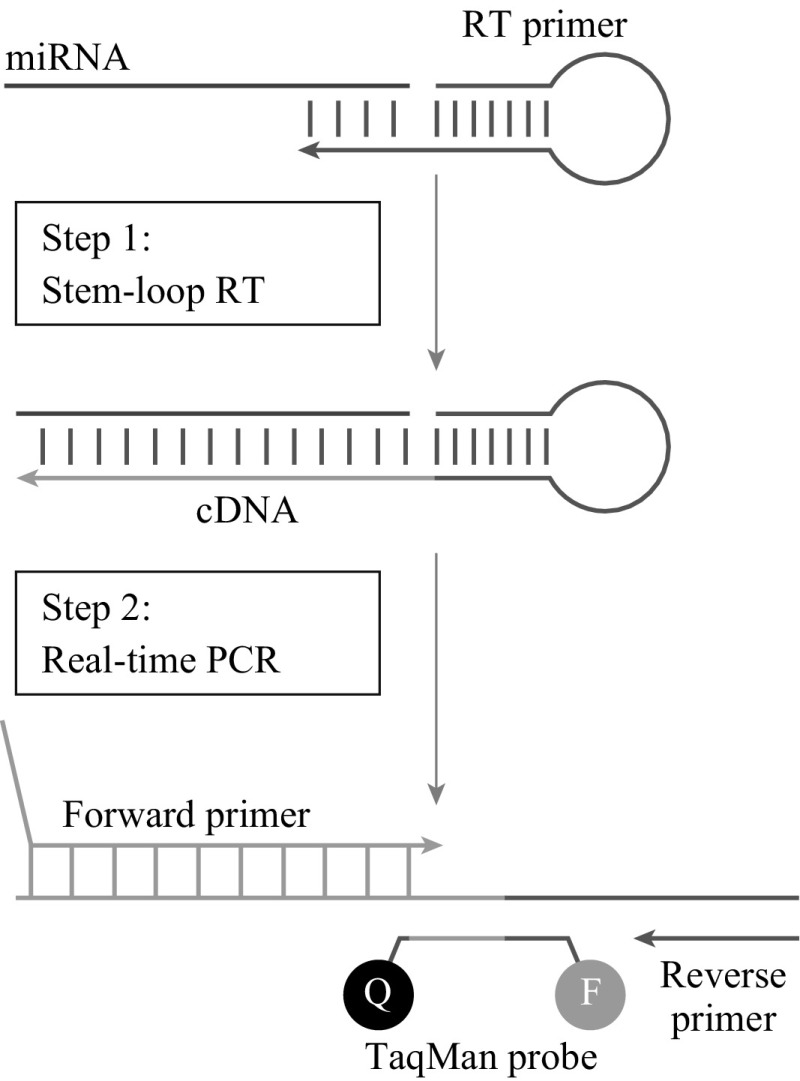
Schematic of RT-PCR in detection of miRNA. Stem-Loop RT primers bind to the 3′ end of miRNA and reverse transcribe the molecule. Then, traditional PCR transcribes the 5′ end. Image taken from Calfu et al. [121]

Polymerase chain reaction (PCR) is one of the most commonly used lab techniques for to analyze DNA samples. This technique can be used for DNA sequencing [78], analyzing the functionality of genes [79, 80], and diagnosing hereditary cases of breast cancer [52, 81–83]. To amplify DNA using PCR, the following are required: two 3’ primers for the strands of DNA, DNA polymerase to synthesize strands of DNA, buffer solutions, and cations, and deoxynucleotide triphosphate. PCR works under the principal of heat cycling, where 30-40 heat cycles are used to denature and rebuild the DNA sequence. The methodology for PCR is as follows: the mixture is heated to 96–98 °C for 20–30 s to denature the bonds between the DNA, leaving two single strands. Then the annealing step occurs in which the reaction temperature drops to 55 °C for 20 s. This step needs to take into account the recommended temperature for hybridization of the primer strands. Once annealing begins, the temperature rises to 72 °C to begin the extension step of the DNA. These denaturing, annealing, and extension steps are repeated for 35 cycles. After the 35^th^ cycle, the extension step is maintained for 5 min to ensure that the single strand of DNA has become a double strand. Then a cool down step is performed, where the temperature drops to 15 °C to hold the sample before it is analyzed [84]. PCR can also be used for RNA analysis. This process is called reverse transcription PCR (RT-PCR). Similar to the methods used for DNA, RT-PCR utilizes heat cycles to reverse transcribe RNA strands. The reagents required are slightly different from those for DNA. The materials required for this process can be seen in ref. 83, and the process is as follows: the reaction mixture is incubated at 23 °C for 10 min, then at 42 °C for 30-60 min. The mixture is then heated for 5-10 min at 95 °C in a water bath, then immediately put into an ice bath [84]. The samples are then analyzed. Early detection of breast cancer using PCR has been well-documented [85–88] and provides insight into the gene and receptor involvement of breast cancer patients. PCR can also be used to identify miRNAs that may present themselves when a patient has breast cancer [89–93]. Figure 6 depicts how RT-PCR can be used to detect miRNA samples. While PCR is a powerful tool, it does have limitations. Because of the need to create DNA/RNA strands, there is a chance that an error or mutation can arise during the process, leading to false positive or negative results. Additionally, PCR, like most medical equipment, requires a great deal of power to run, as well as a trained individual to interpret the results.

### Immunohistochemistry **(IHC)**

IHC is a method by which antibodies are detected in the body. IHC is a staining technique used to identify tumor cells in a tissue sample and is often combined with optical-based techniques like FRET or BRET. IHC allows researchers to gather samples and examine them to physically see whether breast cancer is present, without the use of expensive equipment such as MRIs and CT scanners. IHC requires tissue collection, fixation, and sectioning. Collection is done via biopsy or sample drawing. Fixation often involves the use of paraformaldehyde to prevent tissue decay, as well as staining and washing to highlight target tumor cells that are of interest. IHC is an excellent technique to determine the receptor status of a tumor [91, 94, 95]. The major limitation of IHC is the need for a lab environment and microscope to analyze the sample. IHC also requires tissue staining reagents that may be costly. Lastly, IHC requires a trained individual to determine what is actually present on the sample slides. While IHC does not have heavy energy requirements and is easy to conduct in a lab environment, it is not an ideal solution for early breast cancer detection.

## Microfluidics

Microfluidics is emerging as a novel platform for conducting complex, expensive, time-dense, and technically difficult lab procedures on a microchip that overcomes all of these drawbacks [96–99]. Because of its scaled-down size, a microfluidics platform is able to complete complex tasks very quickly with little reagent involvement. This makes it an ideal platform for diagnostic testing because of its high throughput, low time requirement, and high accuracy characteristics [100]. With regards to the reagents, due to the size of the platform, any fluid flowing in a microfluidics chip undergoes laminar flow. This flow can be adjusted with high accuracy, making it possible to control the flow of the fluid to meet any specific requirement of the user [101]. Typical volume sizes for microfluidics tests are in the mL range (equivalent to a drop of blood), and the only drawback is that it cannot identify markers that do not appear in bodily fluid. Microfluidics chips are made on a silicon wafer in a manner similar to semiconductor chips [100]. They can be specified to meet any demand, and their limitation is fluid flow through the chip. Because of the tight packing of the chip, the flow of a liquid is hindered by high pressures in the system and hydrogen bond forces that interfere with laminar flow. Researchers got around this issue by ionizing fluid as it flows through the chamber [100]. A full review of microfluidics and the science behind it is presented here [101].

A microfluidic chip utilizes the basic physical properties of the micron scale: diffusion is used to transport fluid constituents where they need to be, pressure is used to move volumes of liquid into chambers and electro-resistance [101–103] and fluorescence [104, 105] are used to analyze samples in the fluid. Microfluidics platforms are currently being used to detect genetic mutations that correspond to breast cancer, including DNA and miRNA mutations [106–111]. Due to their high throughput and low time requirements, these platforms are able to analyze more sample volume in a shorter amount of time than traditional detection methods. As a result, microfluidics chips that can analyze multiple proteins in blood have been developed to save time and resources. Fan et al. developed a microfluidics chip that can sample a large range of proteins within 10 min after sample collection [112]. This greatly reduces the stress on resources and time, allowing more time to run tests and less time to wait for them. Microfluidics platforms can also be combined with nanoparticles such as quantum dots (a crystal material used to enhance resolution in sample images). This allows for even greater resolution with a short time requirement for tests [113]. A report conducted by Mei Hu et al. utilized microfluidics chips and QDs to measure cancer biomarkers at a detection limit of 250 fM [113]. Another report by Amily Fang-ju Jou et al. discusses using QDs and FRET to detect miRNAs involved with prostate cancer [114]. While this miRNA is not involved with breast cancer, similar techniques can be utilized to detect the miRNAs involved with breast cancer. Jou’s team developed QDs with cadmium selenide (CdSe) and zinc sulfide (ZnS) and combined this with FRET quencher-functionalized nucleic acids, meaning that excitation occurs in the presence of a nucleic acid. Using this technology, they obtained optical detection sensitivity in the pico-molar range [114].

Microfluidic chips have reached the point where they can separate blood from plasma and conduct tests within a single chip using magnetic beads and other nanoparticles [115]. There have been further advancements with tumor cell detection and separation using microfluidics as well [102, 116]. Because of their size, ease of use, and ability to save time, money, and resources, microfluidics can quickly surpass other detection methods when it comes to diagnosing breast cancer. The only drawback they currently face is the lack of a microfluidics system available that can conduct every single laboratory process on a single chip. There have been chips that are able to conduct multiple processes, such as separation and detection, but no chip currently analyzes and displays the information on a read out that anyone can interpret. This is the next logical step to allow microfluidics technology to eclipse mammograms, CT scanners, and MRI machines as the go-to detection tool for breast cancer. With detection limits getting lower and lower, it becomes possible to detect cancer before it enters its later stages and save patient lives using this technology.

## Future Recommendations

With limited resources and infrastructure, and viewing breast cancer as a global disease rather than one that is only endemic to developing nations, practitioners must have the ability to use diagnostic equipment globally with low energy requirements. Microfluidics-based lab-on-a-chip technology needs to develop the capacity to conduct an entire lab operation on one chip, without the use of any other equipment. The markers needed for detecting breast cancer are available through miRNAs, oncogenes, and proteins. The microfluidics platform now needs to utilize these markers and display the results for any user to understand. Future research will need to be conducted to further understand miRNAs and how they form with respect to breast cancer, typical levels for healthy and diseased patients, and how these levels change with respect to disease progression. This knowledge can then be applied to a microfluidics platform and used to diagnose breast cancer with 100 % certainty. It may also be possible to predict the onset of cancer by monitoring these biomarkers in the body and attempt to reregulate them when they enter some altered state. To achieve this utopian diagnostic technology, first and foremost, studies will need to be conducted on how miRNA expression in the body is altered with respect to mutations that lead to cancer. This fundamental knowledge will pave a path toward better understanding of the role of miRNAs in cancer. Based on present knowledge, an educated selection can be made of specific miRNAs that play meaningful roles in breast cancer. Once a set of miRNAs has been developed, testing can begin to determine how expression values change with the onset of breast cancer. However, to make this a more robust and powerful test, oncogenes and proteins should also be incorporated, as these all play roles during the development of breast cancer and can support the diagnostic power of the test. From the knowledge gained from this study, expression levels of miRNAs, oncogenes, and proteins can be used to predict breast cancer onset, and steps can be taken to prevent the disease from becoming terminal.

## Abbreviations

CDC: Centers for Disease Control and Prevention
DSB: Double strand break
ER^+^: Estrogen receptor positive
ER^−^: Estrogen receptor negative
PR^+^: Progesterone positive
PR^−^: Progesterone negative
HER2: Human epidermal growth factor receptor 2
HER2^+^: HER2 positive
HER2^−^: HER2 negative
SLNB: Sentinel lymph node biopsy
AJCC: American Joint Committee on Cancer
DCIS: Ductal carcinoma in situ
LCIS: Lobular carcinoma in situ
TDLU: Terminal duct-lobular unit
BRCA: Breast cancer susceptibility
HR: Homologous recombination
NHEJ: Non-homologous end-joint repair
RNF: RING-type zinc finger family
FANC: Fanconi anemia complementation group
PALB2: Partner and localizer of BRCA2
BPB: BRCA1-PALB2-BRCA2
BRIP1: BRCA1 interacting protein C-terminal helicase 1
FA: Fanconi anemia
RAD51: RAD51 recombinase
SSB: Single strand break
RAD51C: RAD51 homolog C
BARD1: BRCA1-associated RING domain 1
PTEN: Phosphate and tensin homolog
PI3K: 1-Phosphatidylinositol 3-kinase pathway ()
TIC: Tumor-initiating cells
PDCD4: Programmed cell death 4
CT: Computed tomography
MRI: Magnetic resonance imaging
IHC: Immunohistochemistry

## References

[1] Torre LA, Bray F, Siegel RL, Ferlay J, LortetTieulent J, Jemal A (2015). Global cancer statistics, 2012. CA. Cancer J. Clin..

[2] Kolonel LN, Altshuler D, Henderson BE (2004). The multiethnic cohort study: exploring genes, lifestyle and cancer risk. Nat. Rev. Cancer.

[3] Sallis JF, Prochaska JJ, Taylor WC (2000). A review of correlates of physical activity of children and adolescents. Med. Sci. Sports Exerc..

[4] Brownson RC, Boehmer TK, Luke DA (2005). Declining rates of physical activity in the United States: what are the contributors?. Annu. Rev. Public Health.

[5] Kimm SY, Glynn NW, Kriska AM, Barton BA, Kronsberg SS, Daniels SR, Crawford PB, Sabry ZI, Liu K (2002). Decline in physical activity in black girls and white girls during adolescence. N. Engl. J. Med..

[6] Allison KR, Adlaf EM, Dwyer JJ, Lysy DC, Irving HM (2007). The decline in physical activity among adolescent students: a cross-national comparison. Can. J. Public Health.

[7] Marshall SJ, Biddle SJ, Gorely T, Cameron N, Murdey I (2004). Relationships between media use, body fatness and physical activity in children and youth: a meta-analysis. Int. J. Obes..

[8] Sturm R, Hattori A (2013). Morbid obesity rates continue to rise rapidly in the United States. Int. J. Obes..

[9] Ogden CL, Carroll MD, Kit BK, Flegal KM (2014). Prevalence of childhood and adult obesity in the United States, 2011–2012. JAMA..

[10] H. Pan, R.G. Gray, E.B.C.T.C. Group, Effect of obesity in premenopausal ER+ early breast cancer: EBCTCG data on 80,000 patients in 70 trials. J. Clin. Oncol. (Meeting Abstracts), 32(15_ suppl 503) (2014)

[11] Cecchini RS, Costantino JP, Cauley JA, Cronin WM, Wickerham DL, Land SR, Weissfeld JL, Wolmark N (2012). Body mass index and the risk for developing invasive breast cancer among high-risk women in NSABP P-1 and STAR breast cancer prevention trials. Cancer Prev. Res..

[12] Sinicrope FA, Dannenberg AJ (2011). Obesity and breast cancer prognosis: weight of the evidence. J. Clin. Oncol..

[13] Hjern F, Wolk A, Håkansson N (2012). Obesity, physical inactivity, and colonic diverticular disease requiring hospitalization in women: a prospective cohort study. Am. J. Gastroenterol..

[14] Murphy SL, Xu J, Kochanek KD (2013). Deaths: final data for 2010. national vital statistics reports: from the Centers for Disease Control and Prevention, National Center for Health Statistics. Natl. Vital Stat. Syst..

[15] Kochanek K, Murphy S, Anderson R, Scott C (2004). Deaths: Final Data for 2002. National vital statistics reports: from the Centers for Disease Control and Prevention, National Center for Health Statistics. Natl. Vital Stat. Syst..

[16] Carter CL, Allen C, Henson DE (1989). Relation of tumor size, lymph node status, and survival in 24,740 breast cancer cases. Cancer.

[17] Jemal A, Siegel R, Ward E, Hao Y, Xu J, Murray T, Thun MJ (2008). Cancer statistics, 2008. CA. Cancer J. Clin..

[18] Siegel R, DeSantis C, Jemal A (2014). Colorectal cancer statistics, 2014. CA. Cancer J. Clin..

[19] Boyle P, Levin B (2008). World Cancer Report 2008.

[20] A.C. Society, Breast Cancer Survival Rates, By Stage. http://www.cancer.org/cancer/breastcancer/detailedguide/breast-cancer-survival-by-stage

[21] Esebua M, Schatten H (2013). Cell and molecular biology of breast cancer. Histopathology and Grading of Breast Cancer.

[22] Li C, Uribe D, Daling J (2005). Clinical characteristics of different histologic types of breast cancer. Br. J. Cancer..

[23] Korhonen T, Kuukasjärvi T, Huhtala H, Alarmo E-L, Holli K, Kallioniemi A, Pylkkänen L (2013). The impact of lobular and ductal breast cancer histology on the metastatic behavior and long term survival of breast cancer patients. Breast.

[24] Weigelt B, Peterse JL, Van’t LJ (2005). Veer, Breast cancer metastasis: markers and models. Nat. Rev. Cancer.

[25] Stephens PJ, Tarpey PS, Davies H, Van Loo P, Greenman C, Wedge DC, Nik-Zainal S, Martin S, Varela I, Bignell GR (2012). The landscape of cancer genes and mutational processes in breast cancer. Nature.

[26] Nik-Zainal S, Alexandrov LB, Wedge DC, Van Loo P, Greenman CD (2012). Mutational processes molding the genomes of 21 breast cancers. Cell.

[27] Dvinge H, Git A, Gräf S, Salmon-Divon M, Curtis C (2013). The shaping and functional consequences of the microRNA landscape in breast cancer. Nature.

[28] Nakajima H, Ishikawa Y, Furuya M, Sano T, Ohno Y, Horiguchi J, Oyama T (2014). Protein expression, gene amplification, and mutational analysis of EGFR in triple-negative breast cancer. Breast Cancer.

[29] Network Cancer Genome Atlas (2012). Comprehensive molecular portraits of human breast tumours. Nature.

[30] Caestecker KW, Van de Walle GR (2013). The role of BRCA1 in DNA double-strand repair: past and present. Exp. Cell Res..

[31] Robertson L, Hanson H, Seal S, Warren-Perry M, Hughes D (2012). BRCA1 testing should be offered to individuals with triple-negative breast cancer diagnosed below 50 years. Br. J. Cancer.

[32] Tung N, Battelli C, Allen B, Kaldate R, Bhatnagar S (2015). Frequency of mutations in individuals with breast cancer referred for BRCA1 and BRCA2 testing using next-generation sequencing with a 25-gene panel. Cancer.

[33] Tung N, Garber JE, Lincoln A, Domchek SM (2012). Frequency of triple-negative breast cancer in BRCA1 mutation carriers: comparison between common Ashkenazi Jewish and other mutations. J. Clin. Oncol..

[34] Kuusisto KM, Bebel A, Vihinen M, Schleutker J, Sallinen S-L (2011). Screening for BRCA1, BRCA2, CHEK2, PALB2, BRIP1, RAD50, and CDH1 mutations in high-risk Finnish BRCA1/2-founder mutation-negative breast and/or ovarian cancer individuals. Breast Cancer Res..

[35] Powell SN, Kachnic LA (2003). Roles of BRCA1 and BRCA2 in homologous recombination, DNA replication fidelity and the cellular response to ionizing radiation. Oncogene.

[36] Bunting SF, Callén E, Kozak ML, Kim JM, Wong N (2012). BRCA1 functions independently of homologous recombination in DNA interstrand crosslink repair. Mol. Cell.

[37] Brose MS, Smyrk TC, Weber B, Lynch HT, Kufe DW, Pollock RE, Weichselbaum RR (2003). Holland–Frei cancer medicine. Genetic Predisposition to Cancer, Chap. 16.

[38] Long DT, Walter JC (2012). A novel function for BRCA1 in crosslink repair. Mol. Cell.

[39] Cantor SB, Bell DW, Ganesan S, Kass EM, Drapkin R (2001). BACH1, a novel helicase-like protein, interacts directly with BRCA1 and contributes to its DNA repair function. Cell.

[40] Wong MW, Nordfors C, Mossman D, Pecenpetelovska G, Avery-Kiejda KA, Talseth-Palmer B, Bowden NA, Scott RJ (2011). BRIP1, PALB2, and RAD51C mutation analysis reveals their relative importance as genetic susceptibility factors for breast cancer. Breast Cancer Res. Treat..

[41] Buisson R, Niraj J, Pauty J, Maity R, Zhao W, Coulombe Y, Sung P, Masson J-Y (2014). Breast cancer proteins PALB2 and BRCA2 stimulate polymerase h in recombination-associated DNA synthesis at blocked replication forks. Cell Rep..

[42] Rahman N, Seal S, Thompson D, Kelly P, Renwick A (2007). PALB2, which encodes a BRCA2-interacting protein, is a breast cancer susceptibility gene. Nat. Genet..

[43] Bridge WL, Vandenberg CJ, Franklin RJ, Hiom K (2005). The BRIP1 helicase functions independently of BRCA1 in the Fanconi anemia pathway for DNA crosslink repair. Nat. Genet..

[44] Chen P-L, Chen C-F, Chen Y, Xiao J, Sharp ZD, Lee W-H (1998). The BRC repeats in BRCA2 are critical for RAD51 binding and resistance to methyl methanesulfonate treatment. PNAS.

[45] Carreira A, Kowalczykowski SC (2011). Two classes of BRC repeats in BRCA2 promote RAD51 nucleoprotein filament function by distinct mechanisms. PNAS.

[46] Woditschka S, Palmieri D, Duchnowska R, Jassem J, Badve S, Sledge GW, Steeg PS (2013). Overexpression of RAD51 promotes brain metastases from breast cancer. Cancer Res..

[47] Klein HL (2008). The consequences of Rad51 overexpression for normal and tumor cells. DNA Repair.

[48] Park J-Y, Singh TR, Nassar N, Zhang F, Freund M, Hanenberg H, Meetei AR, Andreassen PR (2013). Breast cancer-associated missense mutants of the PALB2 WD40 domain, which directly binds RAD51C, RAD51 and BRCA2, disrupt DNA repair. Oncogene.

[49] Hashizume R, Fukuda M, Maeda I, Nishikawa H, Oyake D, Yabuki Y, Ogata H, Ohta T (2001). The RING heterodimer BRCA1-BARD1 is a ubiquitin ligase inactivated by a breast cancer-derived mutation. J. Biol. Chem..

[50] Lisa D, Daniela S, Simone YS, Julia K, Lisa W (2012). BRCA1-mediated repression of mutagenic end-joining of DNA double-strand breaks requires complex formation with BACH1. Biochem. J..

[51] Parsons R, Simpson L, El-Deiry WS (2003). PTEN and cancer. Tumor Suppressor Genes Volume 1: Pathways and Isolation Strategies.

[52] Li J, Yen C, Liaw D, Podsypanina K, Bose S (1997). PTEN, a putative protein tyrosine phosphatase gene mutated in human brain, breast, and prostate cancer. Science.

[53] Weng L-P, Smith WM, Dahia PL, Ziebold U, Gil E, Lees JA, Eng C (1999). PTEN suppresses breast cancer cell growth by phosphatase activity-dependent G1 arrest followed by cell death. Cancer Res..

[54] Depowski PL, Rosenthal SI, Ross JS (2001). Loss of expression of the PTEN gene protein product is associated with poor outcome in breast cancer. Mod. Pathol..

[55] Carracedo A, Alimonti A, Pandolfi PP (2011). PTEN level in tumor suppression: how much is too little?. Cancer Res..

[56] Song MS, Salmena L, Pandolfi PP (2012). The functions and regulation of the PTEN tumour suppressor. Nat. Rev. Mol. Cell Biol..

[57] Arafa E-SA, Zhu Q, Shah ZI, Wani G, Barakat BM, Racoma I, El-Mahdy MA, Wani AA (2011). Thymoquinone up-regulates PTEN expression and induces apoptosis in doxorubicin-resistant human breast cancer cells. Mutat. Res./Fundam. Mol. Mech. Mutagen..

[58] Bononi A, Bonora M, Marchi S, Missiroli S, Poletti F, Giorgi C, Pandolfi P, Pinton P (2013). Identification of PTEN at the ER and MAMs and its regulation of Ca2&plus; signaling and apoptosis in a protein phosphatase-dependent manner. Cell Death Differ..

[59] Unseld M, Chilla A, Pausz C, Breuss J, Schabbauer G, Prager G (2014). PTEN dependent angiogenesis is mainly regulated by (tumor secreted-) uPAR. Cancer Res..

[60] Mondal S, Subramanian KK, Sakai J, Bajrami B, Luo HR (2012). Phosphoinositide lipid phosphatase SHIP1 and PTEN coordinate to regulate cell migration and adhesion. Mol. Biol. Cell.

[61] Tamura M, Gu J, Matsumoto K, Aota S-I, Parsons R, Yamada KM (1998). Inhibition of cell migration, spreading, and focal adhesions by tumor suppressor PTEN. Science.

[62] Yamada KM, Araki M (2001). Tumor suppressor PTEN: modulator of cell signaling, growth, migration and apoptosis. J. Cell Sci..

[63] Ma L, Teruya-Feldstein J, Weinberg RA (2007). Tumour invasion and metastasis initiated by microRNA-10b in breast cancer. Nature.

[64] Iorio MV, Ferracin M, Liu C-G, Veronese A, Spizzo R (2005). MicroRNA gene expression deregulation in human breast cancer. Cancer Res..

[65] Yan L-X, Huang X-F, Shao Q, Huang M-Y, Deng L, Wu Q-L, Zeng Y-X, Shao J-Y (2008). MicroRNA miR-21 overexpression in human breast cancer is associated with advanced clinical stage, lymph node metastasis and patient poor prognosis. RNA.

[66] Iorio MV, Casalini P, Piovan C, Di Leva G, Merlo A, Triulzi T, Ménard S, Croce CM, Tagliabue E (2009). microrna-205 regulates HER3 in human breast cancer. Cancer Res..

[67] Yu F, Yao H, Zhu P, Zhang X, Pan Q, Gong C, Huang Y, Hu X, Su F, Lieberman J (2007). let-7 regulates self renewal and tumorigenicity of breast cancer cells. Cell.

[68] Liu R, Wang X, Chen GY, Dalerba P, Gurney A (2007). The prognostic role of a gene signature from tumorigenic breast-cancer cells. N. Engl. J. Med..

[69] Lee Y, Kim VN (2007). In vitro and in vivo assays for the activity of Drosha complex. Methods Enzymol..

[70] Heneghan HM, Miller N, Lowery AJ, Sweeney KJ, Newell J, Kerin MJ (2010). Circulating microRNAs as novel minimally invasive biomarkers for breast cancer. Ann. Surg..

[71] O’Day E, Lal A (2010). MicroRNAs and their target gene networks in breast cancer. Breast Cancer Res..

[72] Sethi S, Ahmad A, Mittal S, Ali R, Chen W, Sarkar FH (2013). Upregulation of miR-10b associated with breast cancer metastasis to brain. Cancer Res..

[73] Frankel LB, Christoffersen NR, Jacobsen A, Lindow M, Krogh A, Lund AH (2008). Programmed cell death 4 (PDCD4) is an important functional target of the microRNA miR-21 in breast cancer cells. J. Biol. Chem..

[74] Gasparini P, Lovat F, Fassan M, Casadei L, Cascione L (2014). Protective role of miR-155 in breast cancer through RAD51 targeting impairs homologous recombination after irradiation. PNAS.

[75] Gøtzsche PC, Nielsen M, Casadei L (2011). Screening for breast cancer with mammography. Cochrane Database Syst. Rev..

[76] de Paredes ES (2007). Atlas of Mammography.

[77] C.E.C. Marie Tartar, M.S. Kipper (eds.). Breast Cancer Imaging: A Multidisciplinary, Multimodality Approach (Mosby Elsevier, Philadelphia, PA, 2008)

[78] Herman JG, Graff JR, Myöhänen S, Nelkin BD, Baylin SB (1996). Methylation-specific PCR: a novel PCR assay for methylation status of CpG islands. PNAS.

[79] Hoffman AE, Zheng T, Yi C, Leaderer D, Weidhaas J, Slack F, Zhang Y, Paranjape T, Zhu Y (2009). microRNA miR-196a-2 and breast cancer: a genetic and epigenetic association study and functional analysis. Cancer Res..

[80] Sgroi DC, Teng S, Robinson G, LeVangie R, Hudson JR, Elkahloun AG (1999). In vivo gene expression profile analysis of human breast cancer progression. Cancer Res..

[81] Paik S, Tang G, Shak S, Kim C, Baker J (2006). Gene expression and benefit of chemotherapy in women with node-negative, estrogen receptor–positive breast cancer. J. Clin. Oncol..

[82] Ford D, Easton D, Stratton M, Narod S, Goldgar D (1998). Genetic heterogeneity and penetrance analysis of the BRCA1 and BRCA2 genes in breast cancer families. Am. J. Hum. Genet..

[83] Reis-Filho JS, Pusztai L (2011). Gene expression profiling in breast cancer: classification, prognostication, and prediction. Lancet.

[84] Innis MA, Gelfand DH, Sninsky JJ, White TJ (2012). PCR Protocols: A Guide to Methods and Applications.

[85] Evron E, Dooley WC, Umbricht CB, Rosenthal D, Sacchi N (2001). Detection of breast cancer cells in ductal lavage fluid by methylation-specific PCR. Lancet.

[86] Ignatiadis M, Kallergi G, Ntoulia M, Perraki M, Apostolaki S (2008). Prognostic value of the molecular detection of circulating tumor cells using a multimarker reverse transcription-PCR assay for cytokeratin 19, mammaglobin A, and HER2 in early breast cancer. Clin. Cancer Res..

[87] Fackler MJ, McVeigh M, Mehrotra J, Blum MA, Lange J, Lapides A, Garrett E, Argani P, Sukumar S (2004). Quantitative multiplex methylation-specific PCR assay for the detection of promoter hypermethylation in multiple genes in breast cancer. Cancer Res..

[88] Fehm T, Hoffmann O, Aktas B, Becker S, Solomayer EF, Wallwiener D, Kimmig R, Kasimir-Bauer S (2009). Detection and characterization of circulating tumor cells in blood of primary breast cancer patients by RT-PCR and comparison to status of bone marrow disseminated cells. Breast Cancer Res..

[89] Cuk K, Zucknick M, Heil J, Madhavan D, Schott S (2013). Circulating microRNAs in plasma as early detection markers for breast cancer. Int. J. Cancer.

[90] Mangolini A, Ferracin M, Zanzi MV, Saccenti E, Ebnaof SO (2015). Diagnostic and prognostic microRNAs in the serum of breast cancer patients measured by droplet digital PCR. Biomarker Res..

[91] Asaga S, Kuo C, Nguyen T, Terpenning M, Giuliano AE, Hoon DS (2011). Direct serum assay for microRNA-21 concentrations in early and advanced breast cancer. Clin. Chem..

[92] Lianidou ES, Markou A (2011). Circulating tumor cells in breast cancer: detection systems, molecular characterization, and future challenges. Clin. Chem..

[93] Kretschmer C, Sterner-Kock A, Siedentopf F, Schoenegg W, Schlag PM, Kemmner W (2011). Identification of early molecular markers for breast cancer. Mol. Cancer.

[94] Jacobs TW, Gown AM, Yaziji H, Barnes MJ, Schnitt SJ (1999). Comparison of fluorescence in situ hybridization and immunohistochemistry for the evaluation of HER-2/neu in breast cancer. J. Clin. Oncol..

[95] Onitilo AA, Engel JM, Greenlee RT, Mukesh BN (2009). Breast cancer subtypes based on ER/PR and Her2 expression: comparison of clinicopathologic features and survival. Clin. Med. Res..

[96] Saadi W, Wang S-J, Lin F, Jeon NL (2006). A parallel-gradient microfluidic chamber for quantitative analysis of breast cancer cell chemotaxis. Biomed. Microdevices.

[97] Hou HW, Li Q, Lee G, Kumar A, Ong C, Lim CT (2009). Deformability study of breast cancer cells using microfluidics. Biomed. Microdevices.

[98] Kwon KW, Choi SS, Lee SH, Kim B, Lee SN, Park MC, Kim P, Hwang SY, Suh KY (2007). Label-free, microfluidic separation and enrichment of human breast cancer cells by adhesion difference. Lab on a Chip.

[99] Van De Vijver MJ, He YD, van’t Veer LJ, Dai H, Hart AA (2002). A gene-expression signature as a predictor of survival in breast cancer. N. Engl. J. Med..

[100] Knight J (2002). Microfluidics: Honey. I shrunk the lab. Nature.

[101] Chen J, Chen D, Xie Y, Yuan T, Chen X (2013). Progress of microfluidics for biology and medicine. Nano-Micro Lett..

[102] Chen J, Li J, Sun Y (2012). Microfluidic approaches for cancer cell detection, characterization, and separation. Lab Chip.

[103] Nguyen TA, Yin T-I, Reyes D, Urban GA (2013). Microfluidic chip with integrated electrical cell-impedance sensing for monitoring single cancer cell migration in three-dimensional matrixes. Anal. Chem..

[104] Song JW, Cavnar SP, Walker AC, Luker KE, Gupta M, Tung Y-C, Luker GD, Takayama S (2009). Microfluidic endothelium for studying the intravascular adhesion of metastatic breast cancer cells. PloS One.

[105] Hsieh AT-H, Pan PJ-H, Lee AP (2009). Rapid label-free DNA analysis in picoliter microfluidic droplets using FRET probes. Microfluid. Nanofluid.

[106] Mangold KA, Wang V, Weissman SM, Rubinstein WS, Kaul KL (2010). Detection of BRCA1 and BRCA2 Ashkenazi Jewish founder mutations in formalin-fixed paraffin-embedded tissues using conventional PCR and heteroduplex/amplicon size differences. J. Mol. Diagn..

[107] Rasooly A, Jacobson J (2006). Development of biosensors for cancer clinical testing. Biosens. Bioelectron..

[108] van Mameren J, Modesti M, Kanaar R, Wyman C, Peterman EJ, Wuite GJ (2009). Counting RAD51 proteins disassembling from nucleoprotein filaments under tension. Nature.

[109] Vorwerk S, Ganter K, Cheng Y, Hoheisel J, Stähler PF, Beier M (2008). Microfluidic-based enzymatic on-chip labeling of miRNAs. New Biotechnol..

[110] Moltzahn F, Olshen AB, Baehner L, Peek A, Fong L, Stöppler H, Simko J, Hilton JF, Carroll P, Blelloch R (2011). Microfluidic-based multiplex qRT-PCR identifies diagnostic and prognostic microRNA signatures in the sera of prostate cancer patients. Cancer Res..

[111] Jang JS, Simon VA, Feddersen RM, Rakhshan F, Schultz DA, Zschunke MA, Lingle WL, Kolbert CP, Jen J (2011). Quantitative miRNA expression analysis using fluidigm microfluidics dynamic arrays. BMC Genomics.

[112] Fan R, Vermesh O, Srivastava A, Yen BK, Qin L (2008). Integrated barcode chips for rapid, multiplexed analysis of proteins in microliter quantities of blood. Nat. Biotechnol..

[113] Hu M, Yan J, He Y, Lu H, Weng L, Song S, Fan C, Wang L (2009). Ultrasensitive, multiplexed detection of cancer biomarkers directly in serum by using a quantum dot-based microfluidic protein chip. ACS Nano.

[114] Jou AF-J, Lu C-H, He Y-C, Ou H, Wang S-S, Hsu S-L, Willne I, Ho JA (2009). Diagnosing the miR-141 prostate cancer biomarker using nucleic acid-functionalized CdSe/ZnS QDs and telomerase. Chem. Sci..

[115] Shim JS, Browne AW, Ahn CH (2010). An on-chip whole blood/plasma separator with bead-packed microchannel on COC polymer. Biomed. microdevices.

[116] Perozziello G, Candeloro P, Gentile F, Nicastri A, Perri A (2014). Microfluidics & nanotechnology: towards fully integrated analytical devices for the detection of cancer biomarkers. RSC Adv..

[117] Moelans CB, van Diest PJ (2012). Breast: Ductal carcinoma. Atlas Genet. Cytogenet. Oncol. Haematol..

[118] Abraham RT (2001). Cell cycle checkpoint signaling through the ATM and ATR kinases. Genes Dev..

[119] Yoshida K, Miki Y (2004). Role of BRCA1 and BRCA2 as regulators of DNA repair, transcription, and cell cycle in response to DNA damage. Cancer Sci..

[120] Khanna KK, Jackson SP (2001). DNA double-strand breaks: signaling, repair and the cancer connection. Nat. Genet..

[121] Chen C, Ridzon DA, Broomer AJ, Zhou Z, Lee DH (2005). Real-time quantification of microRNAs by stem–loop RT–PCR. Nucleic Acids Res..

